# Electroencephalographic and Cardiovascular Changes Associated with Propofol Constant Rate of Infusion Anesthesia in Young Healthy Dogs

**DOI:** 10.3390/ani13040664

**Published:** 2023-02-14

**Authors:** Carla Murillo, Ann B. Weil, George E. Moore, Matthias Kreuzer, Jeff C. Ko

**Affiliations:** 1 Department of Veterinary Clinical Sciences, College of Veterinary Medicine, Purdue University, West Lafayette, IN 47907, USA; 2 Department of Veterinary Administration, College of Veterinary Medicine, Purdue University, West Lafayette, IN 47907, USA; 3 Department of Anesthesiology and Intensive Care, School of Medicine, Technical University of Munich, 80333 München, Germany

**Keywords:** propofol, constant rate of infusion, dogs, EEG, cardiovascular, individual response

## Abstract

**Simple Summary:**

This study aimed to evaluate electroencephalography (EEG) and cardiovascular changes associated with gradual propofol induction followed by three decremental doses of constant rate of infusion (CRI) propofol anesthesia in six young healthy dogs. We found that raw EEG, EEG indices, and mean arterial blood pressure changed significantly according to the anesthetic depth. These values were highly correlated to each other except for heart rate. In addition, individual dog response to propofol can be quantitatively identified with EEG indices in each anesthetic phase. We concluded that EEG monitoring can be used to aid in tracking ongoing brain state changes during different depths of anesthesia. Furthermore, an individual dog’s response to propofol can be quantitatively identified with EEG indices.

**Abstract:**

This study aimed to evaluate electroencephalography (EEG) and cardiovascular changes associated with propofol constant rate of infusion (CRI) anesthesia in dogs. Six dogs were each given propofol CRI to induce different anesthetic phases including induction (1 mg/kg/min for 10 min), and decremental maintenance doses of 2.4 mg per kg per min, 1.6 mg per kg per min, and 0.8 mg per kg per minute over 45 min. Processed EEG indices including patient state index (PSI), (burst) suppression ratio (SR), and spectral edge frequency (95%) were obtained continuously until the dogs recovered to sternal recumbency. The dogs were intubated and ventilated. Cardiovascular and EEG index values were compared between anesthetic phases. The PSI, SR, mean arterial blood pressure, and subjective anesthetic depth scores were highly correlated throughout anesthetic depth changes. The PSI decreased from 85.0 ± 17.3 at awake to 66.0 ± 29.0 at induction, and then sharply reduced to 19.7 ± 23.6 during maintenance and returned to 61.5 ± 19.2 at extubation. The SR increased from 15.4 ± 30.9% at induction to 70.9 ± 39.8% during maintenance and decreased to 3.4 ± 8.9% at extubation. We concluded that EEG indices can be used to aid in tracking ongoing brain state changes during propofol anesthesia in dogs.

## 1. Introduction

Propofol is a commonly used general anesthetic in humans and animals. It has a fast onset of anesthetic action and a relatively short duration of recovery [1]. Due to this reason, propofol is frequently used as the induction agent before inhaled anesthesia, as part of total intravenous anesthesia (TIVA), or as a single agent anesthetic in humans and dogs for minimally invasive surgeries or diagnostic procedures [1,2,3,4,5,6].

While propofol induces dose-dependent brain state changes ranging from sedation to general anesthesia [7,8,9,10,11,12,13], it also induces paralleled dose-dependent hemodynamic and respiratory depression including hypotension and apnea [14]. Current assessments of the anesthetic level of an anesthetized canine patient are mainly based on various surrogate signs of the central nervous system (CNS) that reflect the degrees of anesthetic-induced depression including the animal’s heart rate, blood pressure, respiration, muscle relaxation, and response to nociception with or without purposeful movement [15]. Other information used to assist assessment of the anesthetic level, sometimes also called “depth of anesthesia”, may include the trend in the end-tidal inhalant anesthetic concentrations or pharmacokinetic estimation of the drug duration. While these techniques allowed us to manage anesthesia monitoring safely for years, they lack direct quantitative measures of the ongoing brain state changes and degrees of anesthetic effects on CNS depression in real time. Furthermore, they could not quantitatively differentiate individual patient responses to a given anesthetic drug or protocol. 

Traditionally, EEG has been used widely by neurologists for monitoring brain diseases, including epilepsy. Recent advanced development allows anesthesiologists to use electroencephalography (EEG) for monitoring the depth of anesthesia of their patients in the surgical theater and recovery room [7,8,9,11,12,16]. Based on these EEG findings, excessive levels of anesthesia and certain anesthetics are linked to postoperative delirium in elderly patients, especially those in the geriatric population with cognitive impairment [17]. In addition, using EEG waveform signature markers derived from frontal EEG in real time allows anesthesiologists to titrate anesthetic dosages based on the brain state of the anesthetized patient and avoid excessive or inadequate anesthesia [7,8,9,11,12].

Using EEG for routine monitoring is yet to be standard in veterinary anesthesia because the EEG monitor that can be used to track brain state changes in real time is yet to be identified. Furthermore, previous attempts to use bispectral index monitors in dogs showed mixed results and were difficult to apply clinically [18,19,20,21,22,23,24]. The availability of Sedline^®^ EEG monitors allows clinical monitoring of anesthesia in horses subjected to surgery [25,26,27]. This modern EEG monitoring system has not been assessed in anesthetized dogs. Furthermore, the EEG patterns of specific anesthetic drugs, such as propofol, have been well identified in humans, but have not been explored in dogs in detail. Therefore, the objectives of this study aimed to (1) assess the use of EEG to track brain state changes in dogs during propofol anesthesia, (2) characterize EEG patterns associated with a constant rate of infusion of propofol induction and maintenance, and (3) compare and correlate the processed EEG indices with the traditional cardiovascular and clinical monitoring under propofol anesthesia. 

We hypothesized that the modern EEG monitoring technique can be applied in real time to track ongoing brain state changes in propofol-anesthetized dogs. We also hypothesized that there was a strong correlation between EEG indices and cardiovascular variables during propofol anesthesia.

## 2. Materials and Methods

### 2.1. Animals

Six purpose-bred 16-month-old male intact beagle dogs, weighing 10–13 kg, were used in the study. All dogs were determined to be healthy based on physical exam and laboratory blood work evaluations. Complete blood work, urine analysis, and electrocardiograms were obtained before the beginning of the experiment. Food was withheld for 8 h but the water was free to access before the anesthesia. This study was approved by the Purdue University Animal Care and Use Committee. Dogs were adopted after the experiment.

### 2.2. Instrumentation and Data Collection

In order to administer propofol and balance electrolyte fluids during the study, a cephalic venous catheter was placed in each dog immediately before the study. The Sedline^®^ adult adhesive electrode is only available in one size. To accommodate different sizes of the dog’s head, an alligator clip was used to connect the regular EEG needle electrode (Neuroline subdermal 12 × 0.4 mm–0.5-inch × 27 gauge) with each of the Sedline^®^ adhesive electrode in the position of L1, L2, R1, R2, ground CB, and reference CT lead. This flexible electrode setup allowed consistent positioning of the electrode for connection according to the anatomic landmark without being limited by the size of the original Sedline^®^ electrode. The same electrode setup was employed successfully in our previous EEG study in horses [27]. Before each study, the electrical signal and impedance of each electrode was checked and accepted by the Sedline^®^ monitor automatically. The values of the acceptable electrode impedance range from 0.0 to 65.0 kilo-ohms [28]. Once the electrode is accepted by the Sedline^®^ monitor, a green icon appeared on top of the screen to indicate the position and the acceptance of that particular electrode. If the electrode was rejected by the Sedline^®^ monitor, then a new electrode was used, and the validation process repeated until all the six needle electrodes were accepted by the monitor. 

**Figure 1:**
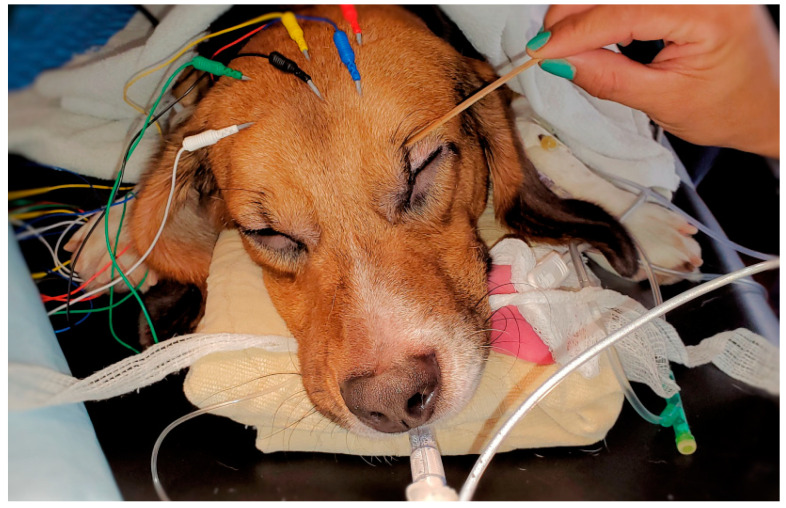
The image shows the position of the color-coded needle EEG leads used in this study. The electrode R1 (white color) was positioned at the Fp2 in the human 10–20 EEG system. The R2 lead (green color) was positioned between F4 and F8 location. The L1 (blue color) lead was positioned at the Fp1 location, and the L2 lead (red color) was positioned between F3 and F7 location. The ground (CB, yellow color) and the reference (CT, black color) electrodes were placed on the mid-sagittal line in the central and the cranial position, respectively. The image also shows the use of a cotton swab for assessing the dog’s eye reflexes (palpebral and globe position).

The placement of the needle electrodes and their related positions according to human EEG 10–20 system were similar to our previous equine study [27]. For R1 Sedline^®^ electrode, it was positioned at the Fp2 on the human 10–20 EEG system position. For the R2 electrode, it was positioned between F4 and F8 location. For the L1 electrode, it was positioned at the Fp1 location. For the L2 electrode, it was positioned between F3 and F7 location. Finally, for the ground (CB) electrode, it was placed on the mid-sagittal central line position, and for the reference (CT) electrode, it was positioned on the cranial location along the mid-sagittal line (Figure 1). These subdermal needle electrode placements were simple and easy. Furthermore, it required no hair clipping or shaving, especially for the short-haired dogs in this study. The needle electrodes were easily maintained in their positions throughout the entire study with only occasionally application of alcohol to enhance needle conductivity subdermally. 

Five minutes before initiation of the propofol induction, baseline systolic, diastolic, mean blood arterial pressure, heart rate, limb-lead electrocardiogram (ECG), and awake EEG were obtained. A clicker was used to assess the dog’s auditory response to the noise and confirmed their responsive behavior with the head turning toward the side of the noise direction. Anesthesia was then induced with propofol (Diprivan^®^ 10 mg/mL) using a constant rate of infusion at a rate of 1 mg/kg/min to capture the loss of consciousness and its associated EEG and behavioral changes. The doses of propofol used in this study were determined based on our pilot study. For anesthetic maintenance, three dosages of propofol were delivered via constant rate of infusion using a syringe pump in decremental order for 45 min with 15 min for each dose. The dosages of propofol were 2.4 mg/kg/min, 1.6 mg/kg/min, and 0.8 mg/kg/min. After the completion of the 0.8 mg/kg/min dose infusion, the dogs were allowed to recover to capture the EEG changes during the emergence from unconsciousness. All the dogs received a balanced electrolyte fluid (Plasmalyte^®^) at a rate of 5 mL/kg/h during the study.

During the propofol induction, eye reflexes (palpebral and corneal reflexes), relaxation of jaws, limb muscle tone, signs of paradoxical excitement, and the first appearance of apnea before endotracheal intubation were assessed for every dog. Once the dog was judged to be ready for endotracheal intubation, the time was noted, and intubation was completed. The dogs were subsequently placed on mechanical ventilation and breathed 100% oxygen with end-tidal CO_2_ maintained between 35 and 45 mmHg until the end of the study when the dog showed return of spontaneous breathing and clinical signs (swallowing, coughing against the endotracheal tube) ready for extubation. 

Standard anesthesia monitoring including heart rate (obtained from electrocardiogram and pulse oximetry), end-tidal CO_2_, indirect systolic (SBP), diastolic (DBP), and mean arterial pressure (MBP), rectal temperature, respiratory rate, and hemoglobin saturation of oxygen were obtained with a multifunction monitor (Digicare LW9XVet, Lifewindow, Digicare Biomedical) at 3 min intervals throughout the anesthesia. A subjective depth score of anesthesia (Appendix A) was also used to assess the depth of anesthesia every 3 min during the study. 

To simulate nociception during the anesthesia, transcutaneous electrical nerve stimulation was performed in the anesthetized dogs with a nerve stimulator attached via two 25-gauge needles inserted in the skin over the lateral aspect of the tibia. The needles were placed 5 cm apart and attached to the nerve stimulator electrode. The device was used in tetanus mode to provide a 0.22-millisecond square wave pulse stimulus of 400 V at intensity settings of 0 to 100 pulses/s (Hz), where 1 indicated the lowest intensity setting (0 Hz) and 9 indicated the highest intensity setting (100 Hz). This method was used in our previous study and was found to be reliable at assessing the antinociceptive property of the anesthetic protocol [29].

At each time point, the nociceptive stimulation was performed for 2 s beginning with the lowest setting. The setting at which there was a response (i.e., gross purposeful movement, including limb withdrawal, head or neck movement, tail twitching, swallowing, or blinking) was determined and recorded. If a test result was negative, the intensity was increased by 1 setting until a response was detected or the maximum stimulation was reached [29]. Both the physiological and antinociceptive data were obtained in 3 min intervals with a physiologic assessment before the antinociceptive assessment.

### 2.3. EEG Measurement and Data Analysis

For the sake of data analysis, the EEG, cardiovascular, and antinociceptive data were divided into 7 time phases (0–6) and compared. The seven phases were Phase 0: awake baseline, Phase 1: anesthetic induction, Phase 2: high dose of propofol CRI (2.4 mg/kg/min), Phase 3: a moderate dose of propofol CRI (1.6 mg/kg/min), Phase 4: low dose of propofol CRI (0.8 mg/kg/min), Phase 5: recovery until extubation after the termination of propofol CRI, and Phase 6: recovery after extubation until the dog achieved sternal recumbency. 

The recorded EEG data were stored automatically by the monitor and could be retrieved via a software (Masimo^®^ Trace^TM^) as CSV files at will. The CSV files displaced as Excel data which contained all the processed EEG indices in high resolution (every 2 s data). These indices include the patient state index (PSI, with 0 as total cortical silence and 100 as complete awake states), burst suppression ratio (SR in %), the electromyography activity (EMG in %), the 95% of spectral edge frequency (SEF_95_) of the left and right hemispheres, and the artifact activity (ART in %). The Sedline^®^ monitor calculated the SR% as the percentage of epochs in the previous 63 s where the EEG silent duration is longer than 0.5 s and the EEG voltage is within +5 to − 5 μV during this period [30]. 

To visual inspection of the raw EEG recording, an edf file was downloaded from the monitor for such a purpose. The two-second EEG data were used for the statistical analysis, providing more than 20,000 data points for some variables. For the SEF_95_ data, the left and right sides of the hemisphere were calculated separately and compared using the paired *t*-tests for hemispheric coherence. The PSI, SR, SEF_95_, EMG, and ART data for each dog was pooled by phase, and then means compared by phases using linear mixed model analyses with repeated measurements. A *p*-value < 0.05 was considered statistically significant. If phase data differed significantly overall for a particular variable, then the results of each phase were compared pairwise with Bonferroni correction/adjusted for multiple comparisons. Data were presented with mean ± SD for both hemodynamic and EEG indices. The Spearman rank correlation coefficient (**ρ**, “rho”) for nonparametrically distributed data was used to assess the correlation between the mean values of the EEG indices and hemodynamic values in each anesthetic phase. PSI, SR, SEF_95_, EMG, ART, HR, SBP, MBP, DBP, and subjective anesthetic depth score were, thus, assessed in pairwise combinations for correlation. 

## 3. Results

### 3.1. Awake Baseline 

The awake EEG waveforms of the dogs were characterized by high frequency (>60 Hz) and low amplitude pattern in all the dogs except one dog that was difficult to obtain due to the temperament of the dog. This dog had a lot of artifacts and greater muscle activity, which masked the original EEG waveform. As a result, this dog’s EEG indices were considered not representative of this phase and were removed from the analysis. The rest of the anesthetic phases of this dog were used in the statistical analysis. 

### 3.2. Endotracheal Intubation Time and Associated Events during the CRI Induction

The dogs were endotracheally intubated between 6 and 10 min after the initiation of the propofol induction. The EEG waveform went from awake pattern of high frequency low amplitude to a lower frequency and higher amplitude pattern during and after induction. All the dogs lost their auditory responses to the clicker noise at the time of endotracheal intubation. Other signs indicating loss of consciousness included sluggishness or lack of palpebral response and loss of jaw tone with minimal tongue withdrawal when the tongue was pulled out of the mouth. Two of the six dogs exhibited paradoxical excitation during the propofol induction, which was characterized by vocalization, non-purposeful movements of the limbs and trunk, struggling and paddling that required physical restraint during the excitation. One of these dogs had a short duration (40~60 s) of excitation and the other dog had a longer duration (~3 min) of excitation. As the propofol gradual induction continued, these excitations subsided spontaneously before the endotracheal intubation. 

### 3.3. EEG Waveforms and Processed EEG Indices

**Figure 2:**
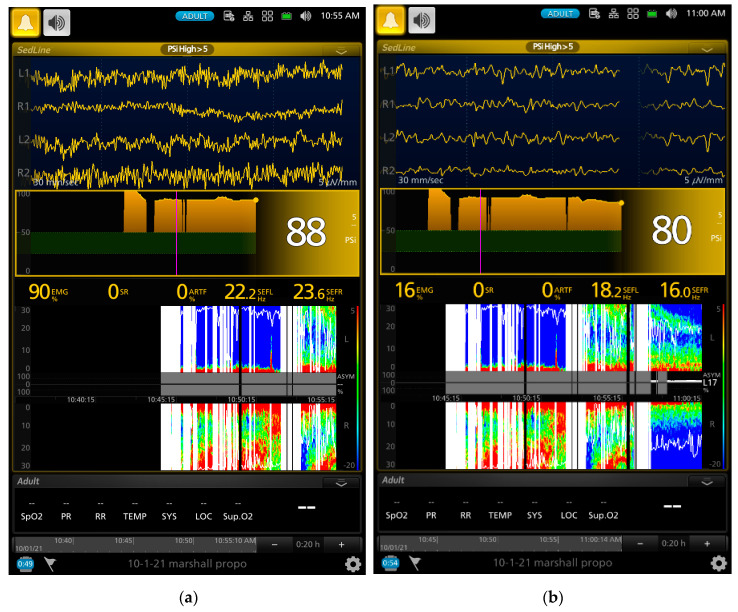
Screenshots of a dog that exhibited paradoxical excitation’s EEG pattern during slow propofol induction. The raw EEG waveforms (the 4 EEG channels in the upper portion of the screen) show regular oscillations of low and high beta waves (**a**). The SEFL_95_ (22.2 Hz) and SEFR_95_ (23.6 Hz) are outlined as the white horizontal lines in (**a**,**b**), which indicate 95% of the waves are below these frequency ranges. The intensity of the spectral patterns of the high and low beta waves (18–30 Hz) appeared on the DSA screen while some intensity of the delta and theta waves appeared in bright red on the DSA screen. The high EMG (90% in (**a**)) indicates a strong muscle activity that can be observed as high-intensity bright spectral color pattern in the DSA. As the propofol induction continued, the EEG patterns shifted from the regular beta waves to large slow oscillation of alpha, theta, and delta EEG patterns (**b**) with SEFR_95_ and SEFL_95_ of 16.0 Hz and 18.2 Hz, respectively. The PSI value also decreased from 88 (**a**) to 80 (**b**) and the EMG value decreased from 90% down to 16%. The color map showed a zipper opening pattern with a reduction in the intensity of high beta toward the alpha spectral pattern as a transition into a deeper plane of anesthesia.

**Figure 3:**
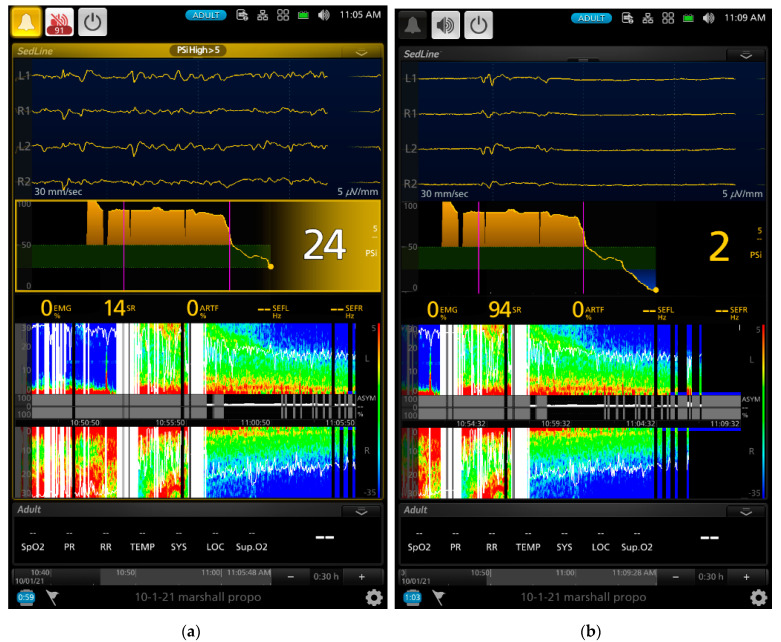
Screenshots of the same dog as presented in Figure 1 in different phases of propofol anesthesia. (**a**) shows a transition of the dog reaching a stable anesthetic plane soon after endotracheal intubation and progressing to a deeper plane of anesthesia. In the figures, the first pink vertical line represents the beginning of the propofol gradual induction, and the second vertical pink line represents the completion of the graduate induction and the beginning of the 2.4 mg/kg/min propofol CRI. The EEG pattern shifted from delta–theta and alpha slow oscillation patterns in (**a**) to prolonged isoelectric flat line EEG wave with burst suppression in (**b**). The spectral pattern of burst suppression appeared on the right side of the DSA as multiple black vertical bars with blue tips in both (**a**,**b**). As the propofol CRI continued, the burst suppression started to become more dominant (from 14% in (**a**) to 94% in (**b**)), and the disappearance of other spectral patterns was due to the prolonged isoelectric EEG pattern. The PSI values fell from the early propofol induction phase of 80 (Figure 2b) to 24 (**a**) and reached a profoundly deep plane of anesthesia with a PSI value of 2 (**a**).

**Figure 4:**
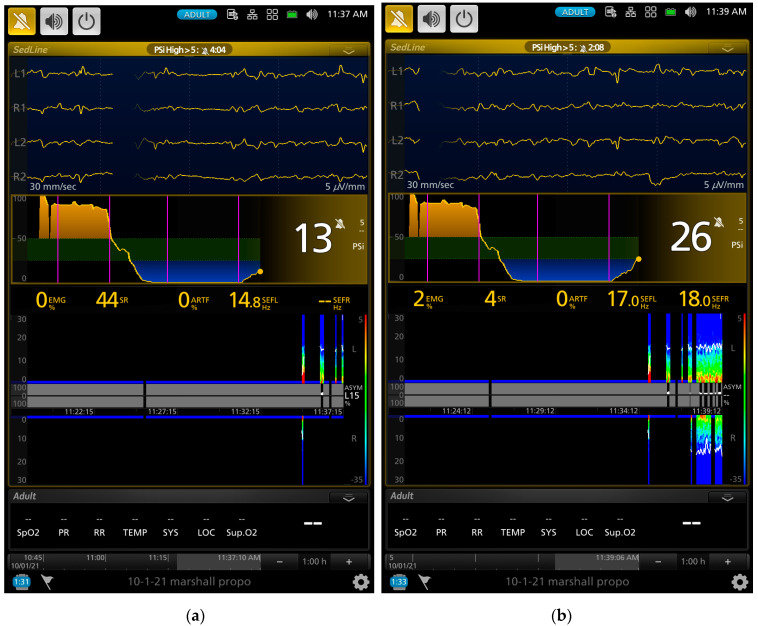
A continuation of EEG screenshots of the same dog shown in Figure 2 and Figure 3. The 4 vertical pink lines (from the left to right of the screen) indicate the initiation of propofol induction, beginning of the 2.4, 1.6, and 0.8 mg/kg/min of propofol CRI, respectively. The trend in the PSI values over time (the middle of the screen) showed the PSI values went from an awake level near 100 to zero during the 2.4 and 1.6 mg/kg/min of propofol CRI and then gradually return to 13 and then 26 when the CRI propofol dosages were reduced to 0.8 mg/kg/min. The raw EEG pattern showed returning of a slow delta–theta wave oscillatory activity (similar to the EEG pattern of Figure 3a) from the previous isoelectric flat line waveform with the occasion of EEG bursts (Figure 3b). The burst suppression ratio value decreased from 44% (**a**) to 4% (**b**). The SEFL_95_ and SEFR_95_ were 17.0 and 18.0 Hz in (**b**).

**Figure 5:**
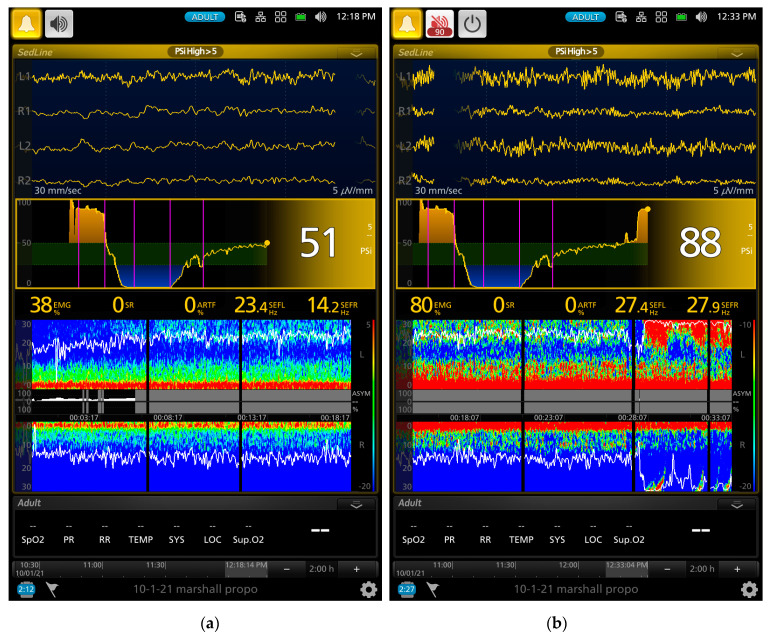
The screenshots of the same dog presented in Figure 2, Figure 3 and Figure 4. The Figures showed the early and late phases of recovery of the dog after the termination of propofol CRI. The 5 vertical pink lines (from the left to right of the screen) indicate the initiation of propofol induction, beginning of the 2.4, 1.6, and 0.8 mg/kg/min of propofol CRI and termination of the propofol CRI, respectively. The EEG waveforms in (**a**) show a transition of continuation of delta–theta (the bright color of the spectrogram) anesthesia and the appearance of low and high beta waves with SEFL_95_ and SEFR_95_ of 23.4 and 14.2. The PSI value increased from the previous 26 to now 51. The PSI trend in (**b**) gives an overview of the entire propofol anesthesia from the awake brain state (yellow shade area on the left side of the trend map) to loss of consciousness after induction, to profound anesthesia with burst suppression and to regain of consciousness in the recovery phase (yellow shade area of the right side of the trend map) to recovery with the PSI value of 88. The high EMG activity (80%) together with high beta power can be seen in the DSA as the bright color between 20 and 30 Hz in (**b**). The SEFL_95_ and SEFR_95_ were 27.4 and 27.9 Hz, respectively. With the change of the DSA scale from (**a**) to (**b**), the alpha power can be seen with delta and theta power in (**b**).

A series of screenshot samples from the EEG monitor of a dog that contained raw EEG waves, density spectral array (DSA, i.e., a color map that provides spectral pattern of the EEG), and processed EEG indices are presented in Figure 2, Figure 3, Figure 4 and Figure 5 during different phases of the propofol anesthesia. Figure 2a,b show the EEG of a dog with paradoxical excitation. It was characterized by a mixture of very regular low and high beta waves (13–30 Hz). These waves are also clearly shown in the spectral power in the DSA, which also coincided with the behavioral manifestation of the dog. 

As the gradual induction with propofol continued, the paradoxical excitation subsided, and the EEG and DSA showed a shift to large slow oscillations of alpha and delta waves (Figure 2b and Figure 3a). As the propofol anesthesia deepened with CRI, the dog became further anesthetized as demonstrated by the low PSI value and dominance of burst suppression with an isoelectric EEG pattern (Figure 3b). A profound anesthesia was reached as indicated by the dominance of burst suppression alternating with prolonged isoelectric flat line of EEG pattern (Figure 3b). When the propofol CRI dose was reduced in the study, the EEG waves of the dog showed delta, theta, and alpha oscillation patterns (Figure 4a) and returned to the moderate plane of anesthesia with the disappearance of burst suppression EEG waveform (Figure 4b). The reappearance of EEG power on the DSA, and increase in the PSI values can be clearly seen in Figure 4a,b. Figure 5a,b show when the propofol CRI was terminated, a mixture of slow wave and non-slow wave EEG patterns appeared with increasing EMG activities. The clinical signs at this time also demonstrated the regain of consciousness with coughing and gaging reflexes and allowed extubation followed by assumption of sternal position and response to the clicker noise. 

The mean values of the processed EEG indices are presented in Table 1. Due to the high resolution (every two seconds during the study) of the EEG indices recorded, many data points (>20,000 for the PSI and SR variables and >10,000 for the SEFL/SEFR) were analyzed. This amount of data resulted in the detection of many statistical differences between anesthetic phases. Therefore, instead of reporting the differences between phases in detail, we chose to report the phases that had statistically non-significant differences for clarity. 

There were significant changes between the seven phases in all the EEG indices. All the values were highly significantly different (*p* < 0.0001) from each other within each index, except the index stated in the following. More specifically, for PSI values, phases 1 and 6 were statistically non-significant from each other, and phases 2 and 4 were statistically non-significant from each other. For SR values, phases 0, 5, and 6 were statistically non-significant from each other. For EMG values, phases 2, 3, and 4 were statistically non-significant from each other, and phases 5 and 6 were statistically non-significant from each other. For ART values, phases 4 and 5 were statistically non-significant from each other. For SEFL values, phase 1 was statistically non-significant from 2 or 6, and phases 2, 3, and 4 were statistically non-significant from each other. For SEFR values, phase 1 was statistically non-significant from 4, 5, or 6, and phases 3 and 4 were statistically non-significant from each other; phases 5 and 6 were statistically non-significant from each other. 

There was a strong correlation between PSI and SR (**ρ** = −0.86, negative correlation due to the same effect with opposite values of these two indices). The MBP was most correlated to anesthetic depth score (**ρ** = 0.76) and then to PSI values (**ρ** = 0.71). The anesthetic depth score was most correlated to the PSI (**ρ** = 0.75) and then to SR (**ρ** = 0.70). The PSI and EMG correlation was 0.52. The correlation between HR and MBP (**ρ** = 0.27), anesthetic depth score (**ρ** = 0.05), PSI (**ρ** = 0.04), and SR (**ρ** = 0.05) were all weak. 

The cardiovascular parameters, subjective anesthetic depth score, and tolerance to electric stimulation data are presented in Table 2. The heart rate, systolic blood pressure, and maximum electrical stimulation values were statistically non-significant between phases. The diastolic blood pressure, mean arterial blood pressure, and anesthetic depth score values were significantly (*p* = 0.01) different between phase 1 (induction) and phase 3 (1.6 mg/kg/min) and the rest of the values were not significantly different between phases within each data category. 

**Figure 6:**
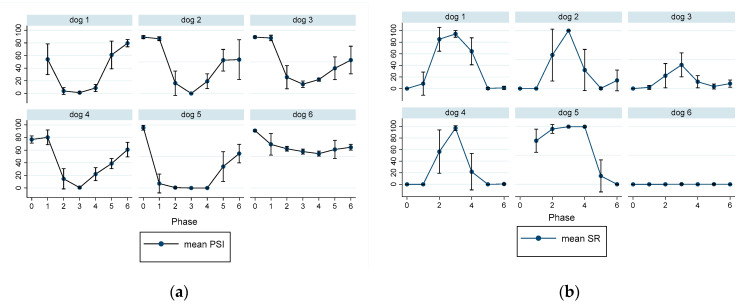
The individual response to propofol was reflected by the different degrees of patient state index (PSI—(**a**)) and burst suppression ratio (SR—(**b**)) across the anesthetic phases in six dogs. The data expressed as mean ± SD for phase 0 (awake), phase 1 (induction), phase 2 (2.4 mg/kg/min), phase 3 (1.2 mg/kg/min), phase 4 (0.6 mg/kg/min), phase 5 (extubation), and phase 6 (recovery to sternal). A shape contrast between dog#5 and #6 in PSI and SR was noticed with dog#5 having much more profound CNS depression indicated by a lower PSI and a higher SR during the same phase of propofol anesthesia than dog#6.

**Figure 7:**
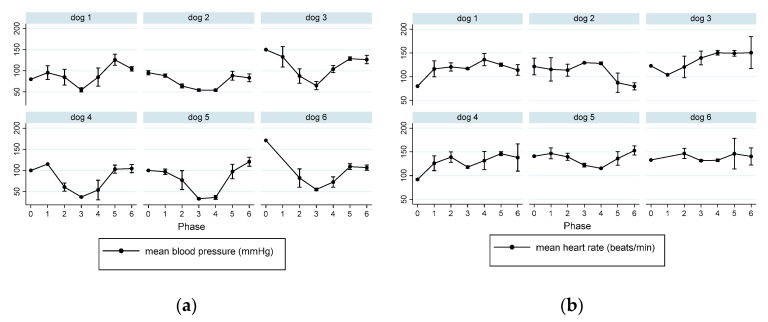
The individual dog’s mean blood pressure (**a**) and heart rate (**b**) responses during each phase of propofol anesthesia. For anesthesia phases, see the Figure 6 legend. The heart rate did not change significantly over the course of anesthesia, whereas mean arterial blood pressure did. However, the MBP responses to propofol between dog#5 and dog#6 were not as noticeable as those observed in EEG indices in Figure 6 between the two dogs.

To illustrate individual dogs’ response to propofol, the data of PSI, SR, HR, and MBP of each dog during the course of propofol anesthesia are presented in Figure 6 and Figure 7. A sharp contrast of the mean PSI and SR trend values is noted between dog#5 and #6, with low PSI values and high SR observed in dog#5 and much less response observed in dog#6 during the same phase of propofol anesthesia. In contrast to this individual difference, a much milder difference in MBP was noticed between the two dogs. Heart rate was not a good indicator of the depth of anesthesia because it did not change much throughout the propofol anesthesia (Figure 7b).

## 4. Discussion

This study was primarily designed as an observational study to determine the feasibility and practicality of using EEG monitoring techniques in propofol-anesthetized dogs. Since each anesthetic drug induces a distinct EEG pattern (propofol vs inhalant or ketamine, for example), instead of using multimodal balanced anesthetic protocol to anesthetize these dogs for the study, the primary goal was to first identify propofol alone-induced EEG features and patterns. Without first identifying each drug signature of the EEG pattern, it is difficult to study the combined EEG patterns when multimodal anesthetic protocols are used in the clinic. 

The results of the current study shows that propofol induced brain state changes in the dog through different phases of general anesthesia, and these brain state changes could be tracked closely with the use of raw EEG and EEG-processed indices together with traditional anesthesia monitoring techniques. Furthermore, the EEG indices can be used to differentiate the individual dog’s response to propofol during anesthesia. 

Propofol is one of the most frequently used general anesthetic drugs. It can be used as an induction anesthetic for endotracheal intubation, or anesthetic maintenance via intermittent boluses or constant rate of infusion in dogs [31]. In clinical practice, propofol is frequently administered as a bolus to achieve rapid induction for endotracheal intubation in dogs [31]. The dose used for rapid sequence induction in healthy dogs without premedication is 6.0 ± 2.0 mg/kg [32]. In the current study, we chose to use a gradual induction with a 1 mg/kg/min constant rate of infusion based on our pilot study, which achieved a state that allowed us to track the potential EEG and brain state changes during the induction. In a recent study, the optimal propofol infusion rate for endotracheal intubation that avoided postinduction apnea in healthy dogs was determined to be 1 mg/kg/min [33]. However, in that study, the dogs were premedicated with intramuscular dexmedetomidine (5 µg/kg) and methadone (0.5 mg/kg) 30 min before the gradual infusion of propofol started. 

The use of the gradual induction technique allowed us to catch propofol-induced paradoxical excitation and its related EEG characters in two dogs in this study. Propofol acts on the brain and spinal cord by enhancing the GABAergic inhibition system [34]. As propofol administration gradually increased in the current study, it produced a progressive increase in the propofol blood concentration and a parallel decrease in the brain’s ability to respond to external stimuli in the dogs. This was evident by the dogs losing their behavioral responses to the clicker noise and progressive loss of their muscle tone with head dropping. While most of the dogs progressed from sedation into general anesthesia, two of the six dogs developed paradoxical excitation, instead of showing progressive signs of being anesthetized. It is well known that the low doses of GABA_A-_potentiating drugs including propofol and midazolam are capable of inducing both behavioral and EEG manifestations of neuroexcitation in humans [35].

Neuroexcitation is an uncommon event but has been reported after propofol administration [36,37]. Such neuroexcitation is defined as an unexpected phenomenon that is characterized by the patient becoming increasingly excited, instead of becoming sedated, with disinhibition and loss of both motor and behavioral control [35]. The loss of motor and behavioral control in children can be characterized by inconsolable crying, combativeness, disorientation, dysphoria, and agitation. A similar description consists of random spontaneous movements, restlessness, talkativeness, hostility, violence, rage, and spontaneous muscle movements of the arms and legs in adults during propofol induction [38]. Similar motor and behavioral responses were observed in the two dogs during their propofol-induced paradoxical excitation. 

The EEG characters of the paradoxical excitation have been described in humans. It is characterized by increasing oscillatory activity with dominant high beta frequency bands (12.5–25 Hz) and decreasing in slow alpha (7.5–12.5 Hz) and theta (3.5–7.5 Hz) frequency bands [39]. The EEG of the two dogs that had paradoxical excitement also had high beta frequency bands. These beta band activities can be seen with the onset and offset in the spectral patterns in the DSA together with the raw EEG patterns in Figure 2 and Figure 3. There is no clear understanding of the propofol-induced neuroexcitation mechanism and no definitive treatments for it. Drug treatments such as sedatives, anticonvulsants, and anticholinergics resulted in unsuccessful attempts in human patients [36,37].

However, a case report in humans using 20% intralipid intravenous bolus followed by CRI resulted in immediate relief of the suspected neuroexcitation of the patient [40]. The authors hypothesized that the intralipid temporarily sequestered the propofol away from the central nervous system and facilitated its distribution to other tissues similar to the treatment of systemic local anesthetic overdose [40]. The two dogs that had neuroexcitation during the induction in this study resolved spontaneously without medical intervention. 

Propofol induces dose-dependent CNS depression [41]. The PSI is an index that the Sedline^®^ monitor uses as a measure of anesthetic levels. The PSI scale of 0–100 corresponds to a patient’s level of sedation/anesthesia. In humans, a PSI value of 100 represents the subject is fully awake [42]. Although we do not know if this PSI scale can be translated directly from humans to dogs, our data in Table 1 show that the PSI values significantly decreased from the awake (phase 0) state of 85.0 ± 17.3 to 66.5 ± 29.0 after anesthetic induction, and then sharply decreased to 19.7 ± 23.6 and 21.0 ± 18.3 during the propofol maintenance (phases 1–4). The PSI value then returned to 48.9 ± 21.5 at extubation (phase 5) and further increased to 61.5 ± 19.2 when the dogs assumed a sternal recumbent position during the recovery (phase 6). The changes in these PSI values were well correlated with the subjective anesthetic depth score, the dog’s clinical response including the electric and clicker noise stimulation, and MBP values. Collectively, this evidence supported that the PSI values can be used to track the brain state changes of these dogs during propofol anesthesia. 

Further evidence supporting the PSI values reflecting the brain state changes of the study dogs are the index of SR and the raw EEG patterns. The SR index is a measure of how much the electrical activity of the frontal cortex of the anesthetized brain is suppressed as a percentage of time in the EEG [43]. The SR index values showed a significant increase as the propofol CRI progressed and reached the peak of suppression with 70.9% ± 39.8% at phase 3. Thereafter, it sharply decreased to 3.4 ± 8.9% during the sternal recovery. The raw EEG patterns shown in Figure 2, Figure 3, Figure 4 and Figure 5 reflect the brain state changes through different phases of propofol anesthesia including awake high-frequency low-amplitude gamma and beta oscillations and then transitioning into delta-theta and alpha oscillations after induction of propofol anesthesia. Subsequently, these high-amplitude, low-frequency waveform patterns changed to burst suppression and prolonged flat isoelectric EEG waves during the profound deep plane of anesthesia. Finally, when the propofol CRI was terminated, the same EEG patterns were observed again in reverse order during the recovery as it occurred from propofol anesthesia back to the awake state. These changes paralleled the PSI values and were highly correlated with the MBP and subjective depth scores in this study. We, therefore, concluded that these processed and raw EEG patterns displayed by the monitor closely reflected the brain state changes of these dogs when anesthetized with propofol in this study. 

It is interesting to note that the blood pressure measurements (systolic, mean, and diastolic) reached the lowest values at phase 3 when the SR reached its peak value (Table 1 and Table 2). The subjective depth anesthetic score also indicated that phase 3 was the deepest phase among all other propofol anesthetic phases. Furthermore, the dogs also did not respond to the maximum electric stimulation during this phase, indicating a lack of response to nociception under this propofol-induced unconsciousness. Collectively, the data from MBP, PSI, SR, the subjective anesthetic depth scores, and the tolerance to electric stimulation of all agreed upon the dogs reaching the deepest plane of propofol anesthesia at phase 3 of this study. 

Propofol produces a dose-dependent reduction in arterial blood pressure in dogs [44]. This negative blood pressure effect was observed in the current study with MBP progressively decreasing after propofol induction and reaching the lowest values when the EEG indices indicated that CNS had the most profound depression at phase 3 of the propofol anesthesia. 

The main purpose of anesthesia monitoring is to prevent a patient from being under or overdosed on anesthetic agents and to protect or treat any potential harm to the patient induced by the anesthetic protocol and surgical side effects. Therefore, any monitoring procedures that can enhance patient safety and are not part of existing monitoring practice standards should be identified and considered to be employed in the future. Current veterinary anesthesia monitoring guidelines focus on the cardiorespiratory surrogate events of the anesthetized patients and are minimally involved in direct monitoring of the central nervous system [15,45]. In the current study, although the EEG indices and MBP are well correlated, the use of MBP and heart rate were less sensitive in identifying individual dogs that had more profound CNS depression than other dogs when receiving the same dose of propofol. The heart rate was not a particularly sensitive indicator of the depth of anesthesia in the current study since it was statistically non-significant between phases of propofol anesthesia. On the other hand, MBP did reflect the different levels of anesthesia more so than HR. When the EEG indices are included as part of the monitoring in the current study, the level of anesthesia could be easily identified. Clinically, the propofol CRI could be titrated to reduce the SR values and confirmed with increased PSI as well as MBP changes in these dogs to improve the quality and safety of the propofol anesthesia. Furthermore, by extracting the information from the EEG indices, individual dog responses to a given dose of propofol can be differentiated as illustrated in Figure 6, and the propofol dose titrated accordingly. 

Another advantage that we observed with EEG monitoring in these dogs was the rapid change in the raw EEG waveforms once the depth of propofol anesthesia changed. This change was much quicker than the blood pressure and can be appreciated by examining the raw EEG waveforms in real time. An example of such change can be appreciated between Figure 3a to Figure 3b, where the raw EEG wave pattern changed to an isoelectric/burst suppression pattern as the anesthesia deepened, and between Figure 4a to Figure 4b, when EEG patterns changed to a higher frequency as the anesthesia lightened. As the dog emerged from propofol anesthesia and regained consciousness, the EEG patterns changed rapidly which can be appreciated in the raw EEG waveforms and the spectrogram in Figure 5a,b.

## 5. Conclusions

In conclusion, in this study, we were able to track the brain state changes associated with propofol anesthesia in dogs with both raw EEG, EEG indices, and spectrogram. These EEG changes were well correlated with MBP and subjective anesthetic depth scores, but with much more rapid speed of assessment in real time. Furthermore, the quantitative degree of CNS depression induced by propofol can be assessed and compared between individual dogs. Further studies should be directed to other anesthetic drugs, other than propofol alone, to see if this EEG monitoring remains valid in dogs. 

## Figures and Tables

**Table 1:** Mean (± SD) EEG index values of six dogs anesthetized with propofol. Propofol anesthesia was divided into awake (phase 0), graduate induction at 1 mg/kg/min (phase 1), 2.4 mg/kg/min CRI (phase 2), 1.6 mg/kg/min CRI (phase 3), 0.8 mg/kg/min (phase 4), recovery to extubation (phase 5), and recovery to sternal recumbency (Phase 6). The indices were PSI (patient state index), SR (burst suppression ratio), EMG (% of electromyographic activity), ART (% of artifact activity), SEFL, and SEFR (95% of the spectral edge frequency on the left and right hemisphere of the cortex). For statistical significance please see the text.

Phase	PSI	SR	EMG	ART	SEFL	SEFR
0	85.0 ± 17.3	0.0 ± 0.0	71.0 ± 33.1	33.8 ± 22.6	24.4 ± 7.1	24.7 ± 8.0
1	66.5 ± 29.0	15.4 ± 30.9	44.8 ± 32.3	18.6 ± 22.2	14.2 ± 7.5	13.4 ± 6.8
2	19.7 ± 23.6	53.6 ± 42.3	6.6 ± 10.3	7.5 ± 15.0	12.8 ± 5.2	14.4 ± 3.8
3	13.0 ± 21.5	70.9 ± 40.0	5.2 ± 7.5	2.8 ± 6.4	12.3 ± 4.7	10.4 ± 5.1
4	21.0 ± 18.3	38.3 ± 40.4	6.4 ± 7.7	0.9 ± 3.3	11.6 ± 5.0	13.2 ± 4.0
5	48.9 ± 21.5	3.5 ± 13.4	27.4 ± 21.5	0.5 ± 3.1	16.9 ± 7.7	13.9 ± 5.2
6	61.5 ± 19.2	3.4 ± 8.9	25.8 ± 24.9	9.3 ± 22.1	13.2 ± 6.9	12.0 ± 6.4

**Table 2:** Cardiovascular parameters (heart rate—HR, systolic blood pressure—SBP, mean arterial blood pressure—MBP, diastolic blood pressure—DBP), subjective anesthetic depth score (depth score), and tolerance to electric stimulation during 7 phases of propofol anesthesia in 6 dogs. The values are presented as mean (± SD). See Table 1 legend for the meaning of all 7 phases.

Phases	HR (bpm)	SBP (mmHg)	MBP (mmHg)	DBP (mmHg)	Depth Score (5-Light, 1-Deep)	Electrical Stimulation (Hz)
0	116.1 ± 23.5	154.4 ± 27.7	111.5 ± 31.5	97.0 ± 34.7	5.0 ± 0.0	NA
1	125.3 ± 21.3	149.5 ± 23.0	101.0 ± 18.3	83.1 ± 18.1	4.2 ± 0.7	900.0 ± 0.0
2	128.8 ± 17.0	124.6 ± 34.0	77.0 ± 18.6	60.9 ± 20.2	2.1 ± 1.0	732.8 ± 244.7
3	126.4 ± 10.2	84.6 ± 16.4	50.6 ± 12.1	36.4 ± 10.8	1.6 ± 0.5	900.0 ± 0.0
4	132.3 ± 13.7	106.7 ± 30.5	67.6 ± 26.3	53.1 ± 23.5	2.3 ± 0.7	880.0 ± 92.5
5	122.4 ± 30.0	146.5 ± 24.1	105.5 ± 19.6	86.9 ± 14.2	3.7 ± 0.9	541.3 ± 270.2
6	126.3 ± 31.5	164.4 ± 21.3	106.7 ± 15.4	88.2 ± 15.6	5.0 ± 0.0	50.0 ± 0.0

## Data Availability

Data is contained within the article.

## Appendix A

**Table A1:** Subjective anesthetic depth score in dogs (Modified from Raue et al., [46]). The palpebral reflex was assessed with a cotton swab and the corneal reflex was assessed with sterile saline drops on the corneal surface via syringe.

Anesthetic Plane	Depth Score	Pupil Size	Globe Position	Eye Reflex (P: Palpebral C: Corneal)	Heart Rate, Blood Pressure, Breathing	Third Eyelid Position	Jaw Tone, Body/Limb Movement, or Swallowing
Very light	5	Large or small, moving	Central	P: Active C: Active	Increased, spontaneous, breathing attempts	Retracted	Strong
Medium–Light	4	Large or small	Central or ventromedial	P: Active or mildly depressed C: Depressed	Increased, spontaneous, breathing attempts	Retracted or partially prolapsed	Medium
Medium	3	Small or medium	Ventromedial	P: Depressed C: Partially depressed	Normal	Completely prolapsed	None or barely
Medium–deep	2	Medium	Central	P: Depressed or markedly depressed C: Depressed	Normal or depressed	Completely or partially prolapsed	None
Very deep	1	Large	Central	P: Absent C: Markedly depressed or absent	Depressed	Partially depressed	None
